# Accuracy of FGF‐21 and GDF‐15 for the diagnosis of mitochondrial disorders: A meta‐analysis

**DOI:** 10.1002/acn3.51104

**Published:** 2020-06-25

**Authors:** Yan Lin, Kunqian Ji, Xiaotian Ma, Shuangwu Liu, Wei Li, Yuying Zhao, Chuanzhu Yan

**Affiliations:** ^1^ Research Institute of Neuromuscular and Neurodegenerative Diseases and Department of Neurology Qilu Hospital Cheeloo College of Medicine Shandong University Jinan Shandong 250000 China; ^2^ Mitochondrial Medicine Laboratory Qilu Hospital (Qingdao) Shandong University Qingdao Shandong 266035 China; ^3^ Brain Science Research Institute Shandong University Jinan Shandong 250000 China

## Abstract

**Objective:**

Given their diverse phenotypes, mitochondrial diseases (MDs) are often difficult to diagnose. Fibroblast growth factor 21 (FGF‐21) and growth differentiation factor 15 (GDF‐15) represent promising biomarkers for MD diagnosis. Herein we conducted a meta‐analysis to compare their diagnostic accuracy for MDs.

**Methods:**

We comprehensively searched PubMed, EMBASE, MEDLINE, the Web of Science, and Cochrane Library up to 1 January 2020. Data were analyzed by two independent reviewers. We obtained the sensitivity and specificity, positive and negative likelihood ratios (LR+ and LR‐), diagnostic odds ratios (DORs) and summary receiver operating characteristic (SROC) curves of each diagnostic method.

**Results:**

Eight randomized controlled trials (RCTs) including 1563 participants (five encompassing 718 FGF‐21 assessments; seven encompassing 845 participants for GDF‐15) were included. Pooled sensitivity, specificity, DOR and SROC of FGF‐21 were 0.71 (95% CI 0.53, 0.84), 0.88(95% CI 0.82, 0.93), 18 (95% CI 6, 54), 0.90 (95% CI 0.87, 0.92), respectively, which were lower than GDF‐15 values; 0.83 (95% CI 0.65, 0.92), 0.92 (95% CI 0.84, 0.96), 52 (95% CI 13, 205), 0.94 (95% CI 0.92, 0.96).

**Interpretation:**

FGF‐21 and GDF‐15 showed acceptable sensitivity and high specificity. Of the biomarkers, GDF‐15 had the highest diagnostic accuracy.

## Introduction

Mitochondrial diseases (MDs) are heritable multisystem metabolic disorders resulting from diverse genetic mutations in nuclear (nDNA) or mitochondrial DNA (mtDNA).^1^, ^2^ MD diagnosis remains challenging even for experienced clinicians due to its wide range of symptoms, particularly in children and the elderly. Effective diagnostics are also lacking, with current MD assessments based on clinical presentation, muscle biopsy, and next‐generation sequencing (NGS).^3^, ^4^ However, these procedures are invasive and time‐consuming. Historically, lactate, creatine kinase (CK), and pyruvate levels in the blood are used for diagnosis, but these markers are nonspecific and lack sensitivity.^5^ Considering the complexity of the diagnostic process, more relevant mitochondrial biomarkers should be identified in the clinic.

Fibroblast growth factor 21 (FGF‐21) regulates lipid and glucose homeostasis.^6^ It is secreted in the liver and functions via binding to cell‐surface FGF receptor (FGFRs) and an essential coreceptor β‐klotho.^6^, ^7^ In 2005,^8^ FGF‐21 was revealed as a metabolic regulator. In 2011,^9^ upon the analysis of 67 patients with MDs, FGF‐21 was shown to be a biomarker. Since its first description, FGF‐21 has attracted intense research attention. Salehi et al.^10^ described it as an indicator to distinguish MDs from other diseases. Morovat et al.^11^ suggested FGF‐21 as a useful tool for MD examinations, particularly in those with chronic progressive external ophthalmoplegia (CPEO). In 2019, Tsygankova et al.^12^ concluded that FGF‐21 levels are elevated in specific metabolic diseases, questioning its reliability as a diagnostic for MDs. The effectiveness of FGF‐21 as an MD marker therefore remains questionable.

Growth differentiation factor 15 (GDF‐15) serves as a TGF‐β family protein that is produced upon detection of inflammation and oxidative stress to maintain tissue homeostasis.^13^, ^14^ In 2014, GDF‐15 was put forward as an MD diagnostic^15^ in TK2‐deficient human skeletal muscle. Similarly in 2015, Yatsuga et al.^16^ highlighted GDF‐15 as a highly specific diagnostic in patients with suspected MDs. In 2016, Davis et al^17^ showed that GDF‐15 outperformed FGF‐21 as a predictor of MD. In 2019, Poulsen and colleagues^18^ further showed the utility of serum GDF‐15 isolated from patients with mitochondrial myopathy to distinguish MD from other myopathy related diseases.

This meta‐analysis was performed to analyze the effectiveness of current MD diagnostics. We comprehensively examined randomized controlled clinical trials to reinvestigate the diagnostic accuracy of FGF‐21 and GDF‐15 for MD patients.

## Methods

The study was carried out following the Preferred Reporting Items for Systematic Reviews and Meta‐Analyses of Diagnostic Test Accuracy Studies (PRISMA),^19^ Meta‐analysis of Observational Studies in Epidemiology (MOOSE)^20^ guidelines, and the Cochrane Handbook for Systematic Reviews of Interventions.

### Database search

PubMed, EMBASE, MEDLINE, the Web of Science and Cochrane Library were reviewed for relevant studies. Trials were published before 1 January 2020 and all publications were written in English. The following terms were used: (“mitochondrial disorders” OR “mitochondrial diseases” OR “mitochondrial myopathies” OR “oxidative phosphorylation deficiencies” OR “respiratory chain deficiency” OR “MDs”) AND (“fibroblast growth factor 21” OR “FGF‐21” or “FGF21”) AND (“growth differentiation factor 15” OR “GDF‐15” OR “GDF15”). Reference lists were employed for the identification of other relevant studies.

### Study inclusion/exclusion

The following inclusion criteria were used: (i) human studies; (ii) participants with MDs or mitochondrial related disease; (iii) FGF‐21 or GDF‐15 used as index tests, muscle biopsy (or genetic diagnosis) as reference standards; (iv) study design: randomized controlled trials (RCTs); (v) studies in which sufficient original data were provided. Specific exclusion criteria were as follows: (i) subjects who were not human beings or patients with MDs; (ii) literature published in the form of review, case report, letter, and commentary; (iii) articles not published in English language; (iv) duplicate publications.

### Data extraction

Data were extracted by two independent researchers. For disagreements, a third researcher reassessed the data to achieve a consensus. For each study, relevant information included: (1) first author and publication year; (2) patients’ number; (3) patients’ mean age and sex ratio; (4) diagnostic accuracy: sensitivity (Sn), specificity (Sp), positive and negative likelihood ratios (LR+ and LR‐), true and false positive (TP and FP), false and true negative (FN and TN). Authors were requested for additional information for studies with incomplete data. If publications stemmed from overlapping sample data, those with the highest number of participants or most detailed information were selected.

### Methodological quality assessment

We evaluated data quality and the risk of bias using QUADAS‐2 (Quality Assessment Tool for Diagnostic Accuracy Studies‐2).^21^ Briefly, QUADAS‐2 consists of four domains, including patient selection, index test, reference standard, and flow and timing. Each domain was assessed in terms of risk of bias (graded as low risk, high risk, or unclear risk), and the first three domains were also considered in terms of applicability (rated as low risk, high risk, or unclear risk). QUADAS‐2 allowed for more objective rating of bias.

### Statistical analysis

Heterogeneity between studies was investigated using Cochran Q and I^2^ statistics. I^2^ values ≥ 50% were considered substantial heterogeneity and a random‐effects model should be used. Otherwise, if I^2^ values＜50% (indicated lower heterogeneity), a fixed‐effects model was applied.^22^ To construct 2x2 tables, information on TP, FP, TN, and FN were recalculated based on the available parameters. The bivariate meta‐analysis model was used to calculate pooled Sn, Sp, LR+, LR‐ and diagnostic odds ratio (DOR).^23^ Based on the Sn and Sp for a single test threshold from each study, the summary receiver operator characteristic (SROC) curve was derived and area under the curve (AUC) calculated. The primary outcome was the diagnostic accuracy of FGF‐21 and GDF‐15 for the diagnosis of MDs, expressed based on Sn, Sp and AUC with corresponding 95% confidence interval (CI). Moreover, to explore the potential sources of heterogeneity, sensitivity analysis and subgroup analysis were carried out. Threshold effects were calculated by testing the correlation coefficient between sensitivity and specificity in the bivariate model, positive values indicated the possibility of heterogeneity. Also, we checked the beta‐coefficient significance in hierarchical summary receiver operator characteristic (HSROC) model, *P* values < 0.05 represent high heterogeneity. For the assessment of publication bias, Deeks' funnel plot was performed.^24^ *P* < 0.05 was the significance threshold. Data were compared using Review Manager 5.3 or STATA 15.0.

### Data availability statement

The corresponding author will provide the data used in this meta‐analysis which are available to qualified investigators upon request.

## Results

### Literature analysis

**Figure 1:**
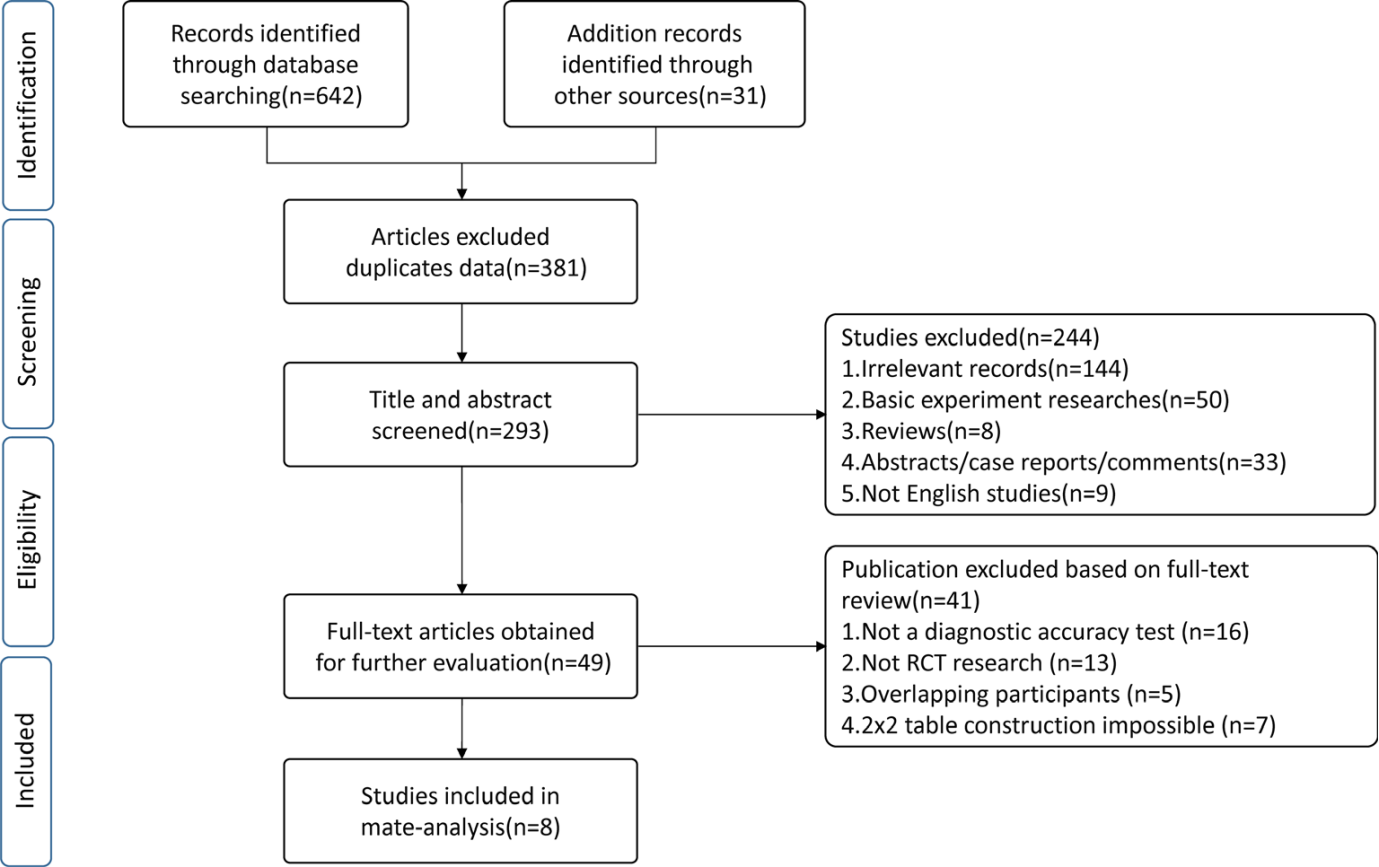
Flow diagram of study selection process. n number of studies.

Databases were comprehensively searched up to 1 January 2020. Data were analyzed by two independent reviewers. Eight RCTs including 1563 participants (five encompassing 718 FGF‐21 assessments; seven encompassing 845 participants for GDF‐15) were included. The initial search yielded 673 references. After screening the titles, 381 were eliminated due to data duplication. After assessment of the titles and abstracts, a further of 244 studies were excluded due to irrelevant records, basic experiments, reviews, case reports comments, and articles not in English. Following text reviews of the 49 articles, 16 were excluded for lacking diagnostic accuracy assessments, 13 were excluded as they were not RCTs, five were excluded for overlapping participants, and seven were removed for 2x2 table construction. Finally, eight studies reached eligibility for subsequent meta‐analysis (Fig. 1).

### Patient characteristics

A total of four studies investigated both FGF‐21 and GDF‐15, a single study investigated FGF‐21, and three studies investigated GDF‐15.^9^, ^12^, ^16^, ^17^, ^18^, ^25^, ^26^, ^27^ Included studies were cohort studies published from 2011 to 2019. Individual studies included 49‐194 cases. In total, FGF‐21 and GDF‐15 were measured via ELISA in 718 and 845 patients, respectively. These studies were carried out in Asia (China and Japan), Europe (Denmark, Finland, Netherlands, Russia, and Spain) and Oceania (Australia). All the assays of FGF‐21 and GDF‐15 were performed in duplicate. The optimal cut‐off values differed for each study and were based on the Youden index (sensitivity + specificity − 1). Basic characteristics are summarized in Table 1.

**Table 1:** Characteristics of the included studies and participants’ baseline demographics.

Study	Country	No. of participants (P/C)	Gender (% males)	Mean age (P/C)	Reference standard	FGF‐21/ GDF‐15	Cut‐off value	Sn (%)	Sp (%)	LR+	LR‐	TP	FP	FN	TN
Koene S et al. (2015)	Netherlands	97/30	29/27	45(spread:17‐72)/ 41(spread:20‐68)	Symptoms and genetic test	GDF‐15	902	52.6 (42.0‐63.0)	96.7 (83.0‐99.2)	15.8	0.5	51	1	46	29
Yatsuga S et al. (2015)	Japan	48/146	48/48	33.6 ± 18.7/ 23.6 ± 13.7	Symptoms,lactate, pyruvate, the lactate/pyruvate ratio, and creatine kinase	FGF‐21	350	77.1 (63.0‐88.0)	87.7 (81.0‐39.3)	6.3	0.3	37	18	11	128
						GDF‐15	710	97.9 (89.0‐99.7)	95.2 (90.0‐98.0)	20.4	0.1	47	7	1	139
Ji X et al. (2016)	China	42/50	60/64	28.8 ± 14.0/ 29.5 ± 12.4	Symptoms, histopathological and/ or genetic tests	GDF‐15	508	97.7 (86.2‐99.8)	92.9 (83.4‐97.3)	13.8	0.1	41	4	1	46
Montero R et al. (2016)	Spain	48/33	gender matched	age matched	Symptoms, biochemical and histopathological tests	FGF‐21	300	52.5 (39.1‐65.7)	96.2 (87.0‐99.5)	13.8	0.5	25	1	23	32
						GDF‐15	550	67.8 (54.4‐79.4)	92.3 (81.5‐97.9)	8.8	0.3	33	3	15	30
Davis RL et al. (2016)	Australia	54/66	total: 42	51.1 ± 13.9/ 40.4 ± 14.4	Symptoms, serum creatine kinase, lactate, and pyruvate levels	FGF‐21	350	68.5 (54.3‐80.0)	91.1 (81.0‐97.0)	7.7	0.3	37	6	17	60
						GDF‐15	2330	77.8 (64.1‐87.5)	94.1 (86.4‐98.8)	13.2	0.2	42	4	12	62
Poulsen NS et al. (2019)	Denmark	28/21	46/48	46(range:17‐70)/ 64(range:26‐70)	Symptoms and histopathological test	GDF‐15	1200	82.0 (63.0‐94.0)	96.0 (76.0‐99.1)	20.5	0.2	23	1	5	20
Tsygankova PG et al. (2019)	Russia	122/60	NA	1 week‐61 years/ 1 month‐60 years	Symptoms and genetic test	FGF‐21	400	51.0 (54.0‐60.0)	76.0 (64.0‐87.0)	2.1	0.6	62	14	60	46
						GDF‐15	children:2400 adult:5000	66.0 (57.0‐75.0)	64.0 (50.0‐75.0)	1.8	0.5	81	22	41	38
Suomalainen A et al. (2011)	Finland	67/74	gender matched	age matched	Symptoms,lactate, pyruvate, the lactate/pyruvate ratio, and creatine kinase	FGF‐21	200	92.3 (81.5‐97.7)	91.7 (84.8‐96.1)	11.1	0.1	62	6	5	68

P, patients; C, controls; Sn, sensitivity; Sp, specificity; LR+, positive likelihood ratio; LR−, negative likelihood ratio; TP, true positive; FP, false positive; FN, false negative; TN, true negative; NA, not available.

### Study quality

**Figure 2:**
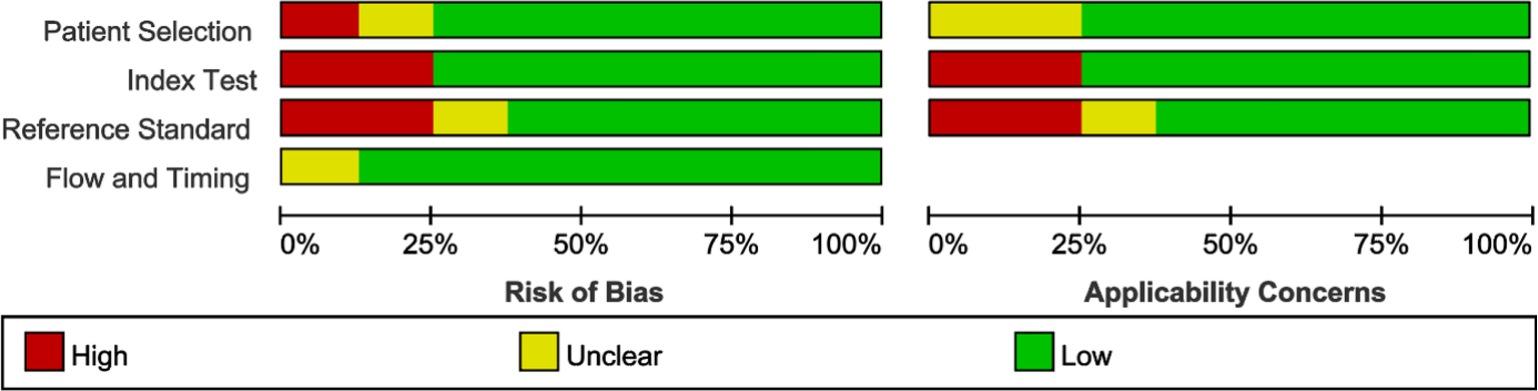
Risk of bias assessment of included studies using the QUADAS‐2 tool. QUADAS‐2, Quality Assessment of Diagnostic Accuracy Studies.

Included studies had a low of bias. Six studies were unbiased in terms of patient selection, six in index tests, and five due to reference standards. Timing and flow showed only one study was unclear and the rest were no bias (Fig. 2).

### Diagnostic accuracy of FGF‐21 for MDs

**Figure 3:**
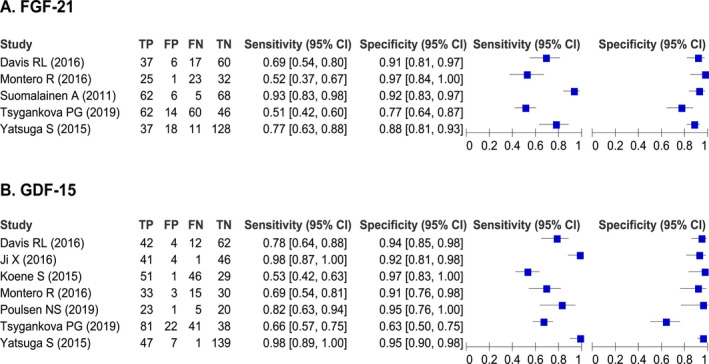
Forest plot of sensitivity and specificity of individual studies for FGF‐21(A) and GDF‐15(B). TP, true positive; FP, false positive; FN, false negative; TN, true negative; 95% CI, 95% confidence interval.

**Figure 4:**
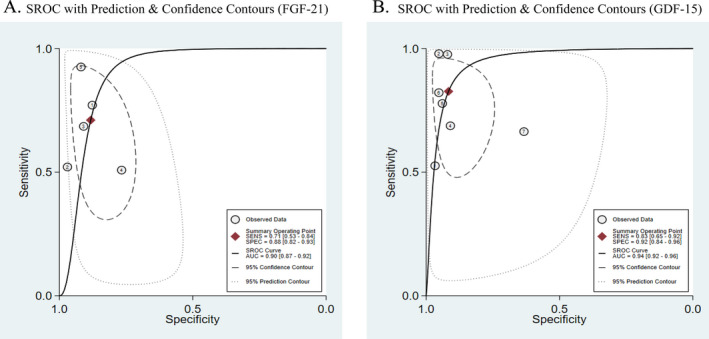
Sensitivity, specificity and summary receiver operating characteristic (SROC) curves of FGF‐21(A) and GDF‐15 (B) in the diagnosis of MDs. A, FGF‐21. *1*: study by Yatsuga S et al.; *2*: study by Montero R et al.; *3*: study by Davis RL et al.; *4*: study by Tsygankova PG et al.; *5*: study by Suomalainen A et al. B, GDF‐15.*1*: study by Koene S et al.; *2*: study by Yatsuga S et al.; *3*: study by Ji X et al.; *4*: study by Montero R et al; *5*: study by Davis RL et al.; *6*: study by Poulsen NS et al.; *7*: study by Tsygankova PG et al. AUC, area under the curve.

Point estimates of FGF‐21 diagnostic accuracy ranged from 0.51 to 0.93 for sensitivity (I^2^ 91.26%, 95% CI 85.23‐97.29) and from 0.77 to 0.97 for specificity (I^2^ 66.31%, 95% CI 34.06‐98.57) across the five studies, with a total of 718 participants (339 patients and 379 healthy controls), (Fig. 3A). Bivariate meta‐analysis produced summary estimates were as follows: sensitivity: 0.71 (95% CI 0.53 to 0.84); specificity: 0.88 (95% CI 0.82 to 0.93); DOR: 18 (95% CI 6–54); AUC: 0.90 (95% CI 0.87 to 0.92). The LR + was 6.10 (95% CI 3.40–10.70); the negative one was 0.33 (95% CI 0.19–0.58). Scatter plots did not appear as “shoulder‐arms” in the SROC curve. Correlation coefficient between sensitivity and specificity in bivariate model was 0.77. Beta‐coefficient significance in HSROC model was −0.86, *p* = 0.263 (Table 2, Fig. 4A).

**Table 2:** Summary of the pooled estimates of FGF‐21 and GDF‐15 in the diagnosis of MDs

	Estimates (95% CI)	
	FGF‐21	GDF‐15
Number of included studies	5	7
Number of subjects	718	845
Sensitivity	0.71 (0.53, 0.84)	0.83 (0.65, 0.92)
Specificity	0.88 (0.82, 0.93)	0.92 (0.84, 0.96)
Positive likelihood ratio	6.10 (3.40, 10.70)	9.90 (4.60, 21.20)
Negative likelihood ratio	0.33 (0.19, 0.58)	0.19 (0.08, 0.42)
Diagnostic odds ratio	18.00 (6.00, 54.00)	52.00 (13.00, 205.00)
AUC	0.90 (0.87, 0.92)	0.94 (0.92, 0.96)

AUC, area under curve; CI confidence interval

### Diagnostic accuracy of GDF‐15 for MDs

Point estimates of the GDF‐15 diagnostic accuracy ranged from 0.53 to 0.98 for sensitivity (I^2^ 92.93%, 95% CI 89.15‐96.71) and from 0.63 to 0.97 for specificity (I^2^ 88.91%, 95% CI 82.16‐95.66) across seven studies with a total of 845 participants (439 patients and 406 healthy controls) (Fig. 3B). Bivariate meta‐analysis produced summary estimates that were 0.83 (95% CI 0.65 to 0.92) for sensitivity and 0.92 (95% CI 0.84 to 0.96) for specificity, and 52 (95% CI 13–205) for DOR, AUC: 0.94 (95% CI 0.92 to 0.96). The LR+ was 9.90 (95% CI 4.60–21.20); negative values were 0.19 (95% CI 0.08–0.42). Scatter plots did not appear as “shoulder‐arms” in the SROC curve. Correlation coefficient between sensitivity and specificity in the bivariate model was 0.36. Beta‐coefficient significance in HSROC model was −0.33, *p* = 0.473 (Table 2 and Fig. 4B).

### Sensitivity analysis and subgroup analysis

**Figure 5:**
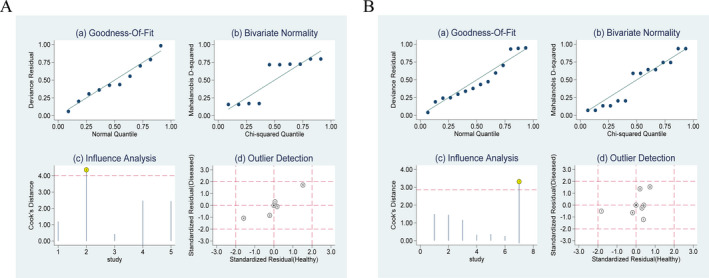
(a) Goodness of fit; (b) Bivariate normality; (c) Influence analysis; (d) Outlier detection. A, FGF‐21. *1*: study by Yatsuga S et al.; *2*: study by Montero R et al.; *3*: study by Davis RL et al.; *4*: study by Tsygankova PG et al.; *5*: study by Suomalainen A et al. B, GDF‐15.*1*: study by Koene S et al.; *2*: study by Yatsuga S et al.; *3*: study by Ji X et al.; *4*: study by Montero R et al; *5*: study by Davis RL et al.; *6*: study by Poulsen NS et al.; *7*:study by Tsygankova PG et al.

Some heterogeneity was evident amongst the included studies in our meta‐analysis. Goodness of fit and bivariate normality suggested that the bivariate random‐effect model was suitable for conduction of the pooled analysis. Influence analysis and outlier detection identified two studies that may overshadow the robustness of the meta‐analysis. (Fig. 5). In order to evaluate the effect of each study on the summary results, we sequentially eliminated individual studies and performed exploratory subgroup analysis for the following factors: ethnicity, sample size, gender and age. Based on the results, we considered that ethnicity and sample size could be the main causes of heterogeneity. The exact values were given in Table 3.

**Table 3:** Subgroup analyses according to the ethnicity, sample size, gender, and age.

	Category	Number of studies	Sensitivity	*P*	Specificity	*P*	LRTChi^2^	*P*
FGF‐21								
Caucasian	Yes	4	0.69 (0.52, 0.87)	0.63	0.89 (0.83, 0.95)	0.09	0.43	0.81
	No	1	0.77 (0.48, 1.00)		0.88 (0.79, 0.97)			
Participants (>150)	Yes	2	0.65 (0.39, 0.91)	0.43	0.84 (0.78, 0.90)	<0.05	4.68	0.10
	No	3	0.75 (0.56, 0.93)		0.92 (0.88, 0.97)			
Gender_matched	Yes	3	0.77 (0.62, 0.93)	0.51	0.91 (0.85, 0.96)	0.14	2.22	0.33
	No	2	0.60 (0.35, 0.85)		0.84 (0.76, 0.92)			
Age_matched	Yes	2	0.77 (0.57, 0.98)	0.73	0.93 (0.88, 0.98)	0.18	3.06	0.22
	No	3	0.66 (0.46, 0.87)		0.86 (0.81, 0.90)			
GDF‐15								
Caucasian	Yes	5	0.69 (0.60, 0.78)	<0.05	0.90 (0.82, 0.98)	0.20	13.32	<0.05
	No	2	0.98 (0.95, 1.00)		0.94 (0.87, 1.00)			
Participants (>150)	Yes	2	0.91 (0.77, 1.00)	0.23	0.85 (0.71, 0.98)	<0.05	4.99	0.08
	No	5	0.78 (0.60, 0.97)		0.94 (0.89, 0.99)			
Gender_matched	Yes	2	0.89 (0.72, 1.00)	0.41	0.94 (0.86, 1.00)	0.96	0.73	0.69
	No	5	0.79 (0.62, 0.97)		0.90 (0.83, 0.98)			
Age_matched	Yes	2	0.89 (0.71, 1.00)	0.46	0.92 (0.82, 1.00)	0.74	0.42	0.81
	No	5	0.80 (0.63, 0.97)		0.91 (0.84, 0.99)			

LRTChi^2^, Likelihood ratio test (chi‐squared test); P < 0.05 indicates statistical significance.

### Publication bias

**Figure 6:**
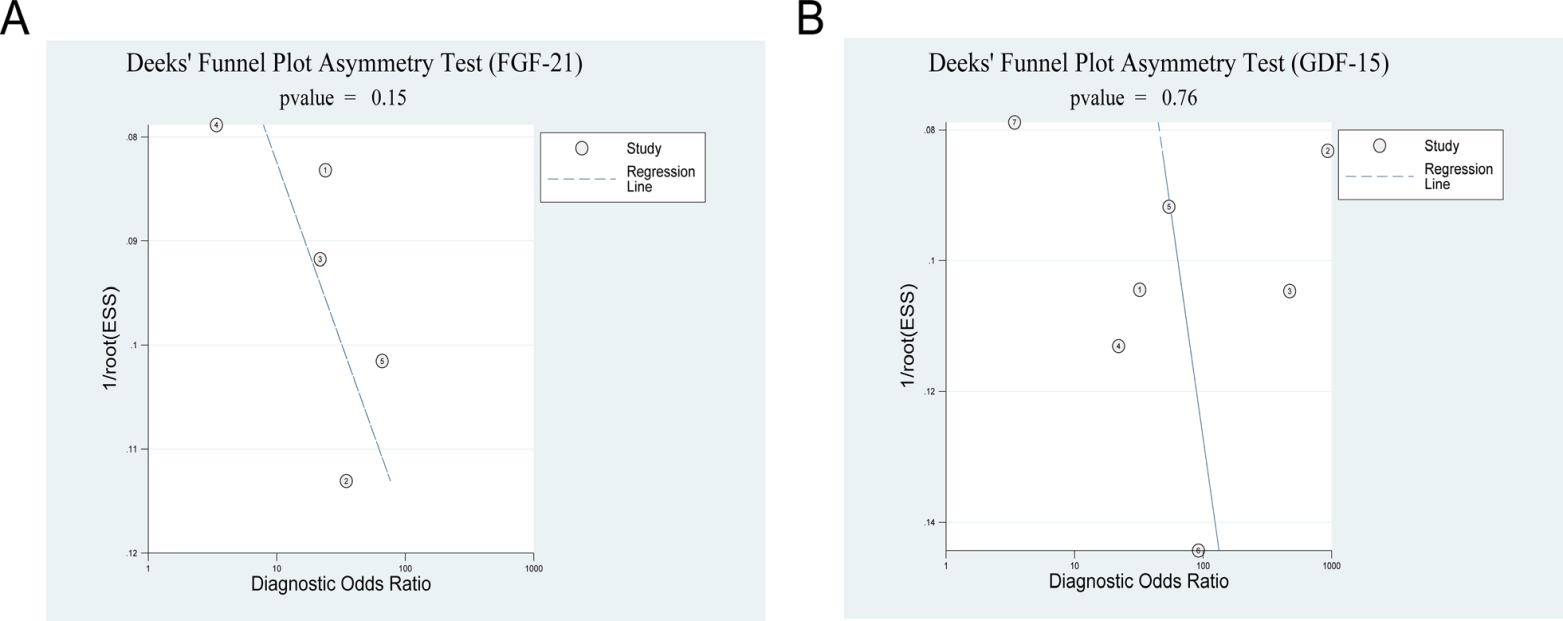
Deeks’ funnel plot for detecting publication bias. A, FGF‐21. 1: study by Yatsuga S et al.; 2: study by Montero R et al.; 3: study by Davis RL et al.; 4: study by Tsygankova PG et al.; 5: study by Suomalainen A et al. B, GDF‐15. 1: study by Koene S et al.; 2: study by Yatsuga S et al.; 3: study by Ji X et al.; 4: study by Montero R et al; 5: study by Davis RL et al.; 6: study by Poulsen NS et al.; 7: study by Tsygankova PG et al.

For bias assessments, Deeks’ funnel plot asymmetry test was used. The results show that the plots were symmetrical indicating minimal publication bias in this meta‐analysis (Fig. 6).

## Discussion

To our knowledge, this is the first meta‐analysis to summarize the current evidence on the diagnostic accuracy of FGF‐21 and GDF‐15 in detecting MDs. An important finding of this work, is that only a small number of articles are available on this topic and the time for publication were relatively new. This could partly reflect FGF‐21 and GDF‐15 being relatively new biomarkers among MDs. Based on the current eight eligible studies, FGF‐21 and GDF‐15 were shown as valid tools for MD diagnosis. Comparison between the two diagnostic indicators showed that GDF‐15 was more sensitive and specific than FGF‐21.

FGF‐21 regulates glucose and lipid homeostasis. In 2000, *Fgf‐21* was documented as the 21st *Fgf* gene.^28^ The function of FGF‐21 was unknown until 2005 upon its identification as a metabolic regulator.^8^ Eleven RCTs now highlight the diagnostic accuracy of FGF‐21 in MDs, but only five were included in this meta‐analysis. This was because we were unable to reconstruct the 2 × 2 tables in five studies^10^, ^11^, ^29^, ^30^, ^31^ and eliminated a single study as it was a follow‐up for one of the included articles.^32^ Our meta‐analysis showed medium sensitivity (0.71) and high specificity (0.88) for FGF‐21. It is known that the AUC and DOR represent overall measures of diagnostic accuracy. The higher the AUC and DOR are, the higher the diagnosis accuracy achieved.^33^ Based on this, the diagnostic utility of FGF‐21 was high (AUC = 0.90; DOR = 18). Other serum biomarkers such as lactate, pyruvate, CK, and lactate‐to‐pyruvate ratio were considered to be nonspecific and lack sensitivity. According to previous research reports,^9^, ^34^ the sensitivity of lactate and lactate‐to‐pyruvate ratio were 63% and 44%, and the specificity was 93% and 100%, respectively. CK levels can be normal or mildly‐to‐moderate elevated with poor sensitivity and specificity in MDs. There is also some evidence which indicates that plasma amino acids, urine organic acids (UOA), and acylcarnitines are useful for the diagnosis of MDs, but the evidence was mostly based on case studies.^35^, ^36^ Our meta‐analysis showed that compared to traditional serum biomarkers, FGF‐21 was superior for the discrimination between MDs and healthy controls.

GDF‐15 is a promising diagnostic biomarker with high sensitivity and reproducibility for MDs and is a TGF‐β cytokine expressed in the central and peripheral nervous systems.^36^, ^37^ Our meta‐analysis summarizes the available evidence of the diagnostic accuracy for GDF‐15 in MDs, including seven RCTs published from 2015 to 2019. The sensitivity, specificity, AUC and DOR for GDF‐15 were estimated as 0.83, 0.92, 0.94 and 52, respectively, as biomarkers for MDs. This highlighted the accuracy of GDF‐15 to predict MDs. GDF‐15 could better reflect the clinical phenotype of MDs, while FGF‐21 showed a higher sensitivity to MDs only when muscle involvement occurred.^17^, ^38^, ^39^ GDF‐15 is thought to be related to disease severity. Yatsuga et al.^16^ investigated serum GDF‐15 levels and MD severity using two mitochondrial scales: the Newcastle Mitochondrial Disease Scale Adults (NMDAS) and Japanese Mitochondrial Disease Rating Scale (JMDRS). Higher GDF‐15 levels were observed as disease severity increased. Koene et al.^30^ evaluated GDF‐15 levels and MD severity in m.3243A>G carriers and reported their correlation with disease severity, but not disease progression. Ji et al.^26^ identified a significant correlation between GDF‐15 and MELAS severity using NMDAS and suggested that GDF‐15 could be used as a reliable indicator to identify MDs severity. GDF‐15 levels in the serum were also related to the proportion of ragged red fibers (RRFs) in the muscle but not COX‐negative fibers.

There was significant heterogeneity existed between the included studies for sensitivity and specificity. By analyzing the correlation coefficient between sensitivity and specificity in bivariate model and HSROC model, we considered that the heterogeneity was acceptable. Exploring the sources of heterogeneity is useful for understanding potential factors that affect the diagnosis accuracy. We used a random‐effects model and carried out exploratory subgroup analysis. The results showed that ethnicity and sample size might be potential sources of heterogeneity. However, due to the small number of studies and lack of necessary data, there were certain limitations in the heterogeneity analysis.

FGF‐21 and GDF‐15 are highly sensitive and specific for MD diagnosis, particularly GDF‐15. In addition to their diagnostic value, other advantages such as reproducibility, safety, cost‐effectiveness, and time‐efficiency must be considered to prove their clinical applicability. Regarding safety, compared with muscle biopsy, FGF‐21 and GDF‐15 were noninvasive and avoided complications (bleeding and infection). Regarding cost‐effectiveness, genetic tests are the gold standard for MD diagnosis, but they are expensive. Less‐costly clinical evaluations should be used to assess the requirement for NGS analysis.

In recent years, it has been reported that GDF‐15 can predict the therapeutic outcomes of mitochondrial treatment. In 2015, Tanaka et al.^40^ used GDF‐15 to assess the efficacy of pyruvate in 2SD hybrid cells (with MELAS‐causing mutations) and control cells, identifying GDF‐15 as a promising therapeutic indicator. In 2019, they enrolled 11 MD patients and confirmed these findings.^41^

The major strengths of this meta‐analysis are the rigorous protocols for the selection and assessment of eligible studies. Due to the relatively strict inclusion criteria, the included studies showed minimal bias and were of high quality. Some limitations should however be noted. Firstly, the small number of studies and sample size did not allow us to explore the data further. Secondly, during the selection process, there were a total of seven observational studies that focused on this topic that were excluded due to incomplete data (unable to construct 2 × 2 tables). We attempted to contact the corresponding authors for their data, but failed. Thirdly, although we evaluated the diagnostic accuracy of FGF‐21 and GDF‐15 for MDs, we did not assess their relationship to clinical phenotype, severity, and progression given the limited number of RCTs and inadequate data. Finally, due to the small number of studies and incomplete data, certain limitations in the heterogeneity analysis has existed in this meta‐analysis. These inconsistencies may mask our data.

In summary, this meta‐analysis was the first to assess the diagnostic value of FGF‐21 and GDF‐15. Considering accuracy, safety, cost, availability, and efficiency, these two cellular factors may be viable biomarkers for MD diagnosis. GDF‐15 seems to outperform FGF‐21 as a diagnostic biomarker. GDF‐15 should therefore be combined with FGF‐21 for first‐line test when MDs are suspected and chosen to prioritize patients for invasive muscle biopsy. Further studies with larger samples are essential to establish the utility of these markers for guiding clinical management decisions and treatment selection.

## Conflict of Interest

The authors declare no conflicts of interest.
